# Pain Coping Strategies for Children with Arthritis

**DOI:** 10.1155/2013/741428

**Published:** 2013-07-17

**Authors:** Kim J. Rosenzweig, Laura Nabors

**Affiliations:** ^1^Department of Psychology, Xavier University, Cincinnati, OH 45207, USA; ^2^School of Human Services, University of Cincinnati, Cincinnati, OH 45221, USA

## Abstract

*Objective*. To present information on pain management strategies for children with juvenile idiopathic arthritis (JIA). *Methods*. The second author developed a manual to present pain management strategies to children. The use of the manual was pilot-tested with a group of children with JIA. Telephone interviews were used to gather information on implementation of pain management strategies. *Results*. Children were able to implement the pain management strategies. Children reported a reduction in daily pain experiences related to JIA when using the pain management strategies. *Conclusions*. The pain management strategies were successful as an adjunctive intervention for short-term pain management. Pain symptoms related to JIA can severely limit children's participation in daily activities. Further study on how children use pain management strategies to improve their involvement in daily activities will provide useful clinical information.

## 1. Introduction

The study of rheumatic illnesses in children is one way in which researchers can learn more about effective interventions for children with chronic pain. Approximately one child per every 1,000 has a rheumatic disease, such as juvenile idiopathic arthritis (JIA) [1]. JIA is the most common form of arthritis in children, has seven subtypes, and has no known cause. This disease can cause changes in the joint(s) such as inflammation and/or stiffness and damage to the joint [2]. The pain associated with this disease occurs in unpredictable flare-ups in which pain and joint swelling can increase. These flare-ups may also cause the child to exhibit fatigue and decreased movement [3]. McGrath and colleagues reported that more than 75% of children with arthritis experience chronic pain [4].

Studies have shown that cognitive-behavioral pain management strategies have been effective in treating children's pain [5, 6]. Central to these theories is the idea that thoughts influence behavior. McGrath describes distraction as the most common method [7]. Parents use this strategy automatically (e.g., diverting a child's attention toward a toy in order to lessen the perception of pain caused by a skinned knee). Another successful strategy is relaxation, such as deep breathing or muscle relaxation, which Rapoff and others found helpful in producing associated reductions in negative emotions and pain [2]. When children use positive self-talk (i.e., provide positive messages to themselves for coping with pain) and actively use imagery (thinking about positive experiences they have had), relaxation, and distraction, they report feeling lower levels of pain [8]. In contrast, negative coping strategies include negative self-talk, a lack of social support network, emotion-based focusing (where children focus on the negative aspects of the pain experience and avoiding active coping while internalizing negative feelings about having pain) and can intensify a child's pain experience [5].

## 2. Materials and Methods

The primary purpose of this brief paper is to review ideas presented in the Change the Channel Manual for Children (*CTC Manual*), which presents cognitive-behavioral strategies that would assist young children with arthritis in coping with their pain [9]. The *CTC Manual* uses the idea of a television and remote control to show children that they can change their pain experience. There is also an explanation of how pain messages travel in one's body. There are five channels to select from: (1) the positive talk channel, (2) the positive thinking channel, (3) the relaxation channel, (4) the doing something else channel, and (5) the tell someone and ask for help channel.

When learning about channel one, children learned to use positive self-talk and to develop their own “pep talk” to help them get ready to use their strategies for coping with pain. For channel two, positive thinking, children practiced developing a superhero to eliminate, minimize, and move their pain (i.e., to use imagery to facilitate coping). Channel three, the relaxation channel, taught children two types of relaxation techniques: breathing exercises and the rock-sponge relaxation technique (be a sponge full of water, tense your muscles, and then release the water to relax the muscles). As they moved to channel four or the distraction channel, children learned to think of positive images, such as things they really enjoy doing (e.g., reading a book, going to the circus, and playing a game) that help to distract them from thinking about their pain. They also learned to enjoy fun things they like to do so that they could “do something else that is fun” when they felt pain. Finally, as they learned about channel five or the support channel, children identified ways to let their parents, teachers, classmates, and friends know about their pain and find ways to tell these support people how they would like to be helped in terms of managing their pain. In a final session, they picked their favorite pain channel and decided under what circumstances they would use different strategies. Key excerpts from this manual are presented in the Supplementary Material Appendix available online at http://dx.doi.org/10.1155/2013/741428.

The next section of this paper reviews information from a pilot project to determine children's perceptions of the strategies. Participants were 13 children, 2 males, and 11 females, between the ages of eight and twelve years, who learned to use the strategies in the *CTC Manual* and reported on their effectiveness in reducing daily arthritis pain on two occasions. Participants were from children's hospitals in the midwestern region of the United States. Parent permission and child assent were required for participation. A university-based institutional review board approved this study.

A child psychologist taught parents and children in the intervention group the strategies in the *CTC Manual* [9]. Parents and children took the manual home and practiced one of the five activities each day for one week: (1) positive statements (self-talk) about coping with pain, (2) positive or mastery-oriented imagery, (3) relaxation, (4) distraction, and (5) ideas for seeking support from teachers and friends. During the time that children and parents practiced the coping strategies, children completed pain diaries during two different telephone interviews during the week that they practiced using their strategies. A pain diary was used to assess change in children's pain. Children were asked to rate how their level of pain changed when using Change the Channel pain management strategies. The rating scales depicted pain as being very high (4) to very low (1).

## 3. Results

Children reported a reduction of their pain when they used the pain management strategies provided in the *CTC Manual *[9]. Strategies were successful in 26 telephone interviews. Specifically, for 12 entries, children reported pain reduction of 2 levels, and there was one entry of a 4-level reduction in pain. Seven entries indicated that pain was reduced by one level and 5 entries indicated a pain reduction of one and a half levels. The mean level of pain reduction in the diaries was 1.59 (SD = 0.73). Children used all of the strategies in the manual to cope with their pain, often combining strategies to help them cope.

## 4. Conclusions

As we learn more about the ways in which children can learn to cope with pain, it is important to continue to conduct further empirical studies to investigate cognitive-behavioral techniques that can assist children in this battle. One in every 1,000 children copes with the chronic pain of JIA [1]. These children want to live their lives as typical children do: going to school, playing sports, and participating in all the events of life. Their JIA-related pain often prevents them from doing so. For this reason, it is important for researchers to continue to investigate the impact of cognitive-behavioral strategies as adjunctive interventions for managing JIA-related pain. It also will be important to investigate what pain management strategies are most effective for children with different subtypes of arthritis. More research about helping children change negative coping strategies into positive ones will assist those children who are having the most difficulty coping with their pain. Further research may give children the tools they need to feel that they have a measure of control over their pain. This is vital in helping them develop positive coping strategies, increase their quality of life, and reduce their pain.

## Supplementary Material

Description of the pain experience
Here is how pain works: The receptors for pain are located in many places, like the skin, muscles, bones and joints. Your arthritis can cause pain messages to start in your joints.
The A-Delta and C nerve fibers will send the pain message and then neurons will turn the pain message into an electrical signal that tells your brain you HURT. What happens is that the A and C fibers take the message up the spinothalamic track to your brain, in places called the thalamus and the frontal cortex. Your brain helps tell you about the pain message.
You can help yourself think away pain. Your brain sends messages back to your body and nervous system that shows how you will cope with pain.
So, your thoughts allow you to turn down the volume on your pain – this means make it LESS or lower. Your thoughts can also help you to forget your pain or CHANGE THE CHANNEL on your pain.
