# Supplementary material for: Pain Coping Strategies for Children with Arthritis
Source: Biomed Res Int. 2013 Jul 17;2013:741428. doi: 10.1155/2013/741428 (PMC3730216; doi:10.1155/2013/741428)
Supplement: Supplementary file 1 — Description of the pain experience Here is how pain works: The receptors for pain are located in many places, like the skin, muscles, bones and joints. Your arthritis can cause pain messages to start in your joints. The A-Delta and C nerve fibers will send the pain message and then neurons will turn the pain message into an electrical signal that tells your brain you HURT. What happens is that the A and C fibers take the message up the spinothalamic track to your brain, in places called the thalamus and the frontal cortex. Your brain helps tell you about the pain message. You can help yourself think away pain. Your brain sends messages back to your body and nervous system that shows how you will cope with pain. So, your thoughts allow you to turn down the volume on your pain – this means make it LESS or lower. Your thoughts can also help you to forget your pain or CHANGE THE CHANNEL on your pain. [file 741428.f1.pdf]

Appendix: Excerpts from the *CTC Manual*

I. Description of the pain experience

Here is how pain works.
The receptors for pain are located in many places, like the skin, muscles, bones and joints. Your arthritis can cause pain messages to start in your joints.

The A-Delta and C nerve fibers will send the pain message and then neurons will turn the pain message into an electrical signal that tells your brain you HURT. What happens is that the A and C fibers take the message up the spinothalamic track to your brain, in places called the thalamus and the frontal cortex. Your brain helps tell you about the pain message.

You can help yourself think away pain. Your brain sends messages back to your body and nervous system that shows how you will cope with pain.

So, your thoughts allow you to turn down the volume on your pain – this means make it LESS or lower. Your thoughts can also help you to forget your pain or CHANGE THE CHANNEL on your pain.

II. There are five channels to help you change the channel and reduce your pain.

Channel 1: Positive Talk

I can say positive things to myself when I have pain. This will help me fight my pain and make it less.

I can beat my pain!

Here are some things I can say…

I can control my pain. I can relax and make it hurt less. I do not have to pay attention to this pain. I can make the pain go down!

What do you want to say to your pain? Use your thoughts to talk back to your pain! Say this inside your head to yourself to help you fight back against your pain. You don't have to say it out loud.

Write down what you would say to yourself to fight arthritis pain…

____

____

Channel 2: Thinking about something that is Fun or Relaxing

You also can do something fun,
by using your imagination!

You can be your favorite superhero beating your pain!
Be a superhero … pick a hero to help you get rid of your pain. Make up a movie to play in your head. In the movie let your hero make your pain go away! Tell your story to your Mother or Father two times. Play this movie in your head when you have a pain flare.

Draw your own superhero right here…what is your hero doing to get rid of your pain?

You can think of your favorite thing to beat your pain!
Trudy's favorite spot is the beach. She thinks about the beach and the ocean and warm sun when she wants a vacation from her arthritis pain. Trudy likes to make sand castles and relax at the beach. She pretends the warm sun makes her pain feel less. She sees a boat in the water and the birds flying over the water. She hears the waves as they hit the sand. Can you see the beach? Can you hear the water? Now, is your pain less?

What is your favorite place?
What is it like?
What happens there?
What does it sound like?

Is it warm or cold?
What do you see?
What are you doing?
What happens next?

Channel 3: Relaxation Channel

Deep breathing can help your body relax and feel better. Pretend you are blowing bubbles. Take a deep breath and slowly let it out (count to 4). Then try this again two more times!

Let's try another relaxation exercise….
First be a rock
Then be a sponge. You can relax and squeeze away the pain.

Let's think about the super relaxation sponge kid. This kid relaxes by practicing making muscles tight like a rock and then relaxing and taking deep breaths. Sponge kid squeezes the pain out when relaxing.
Now, it is your turn practice being the super relaxation kid. Here is what you do.

Sit in a chair.
Make your muscles tight, like a rock.
Then, make them loose, like a sponge sinking in to your chair.
Take some deep breaths and close your eyes.

How do you feel? Is your pain less?

Channel 4: Doing Something Else that is FUN

Did you know you could do something you think is fun so you will not think about your pain?

Here are some fun things…

Read a book!

Play a game with someone you like.
Write down some fun things you can do….

Channel 5: Getting Help from Other People

Other people like teachers can help you when you have a pain flare from your arthritis. You need to ask them for help and make sure that they know how to help you.

Make a list of people who can help you feel better when you have pain.

Now, either you write down or have your parent write down when you can use each strategy.

Name of Pain Channel	When I can use it

Now you are finished… Keep this book in your room at home, so you can use it when you need it. Your parent has a copy too. Remember you can ask them for help too. You should keep using these pain channels whenever you need them. They will help for a long time!
Thank you…you are the best…for dealing with your pain!
